# (*E*)-*N*′-(3,4-Dihy­droxy­benzyl­idene)-2,4-dimethyl­benzohydrazide monohydrate

**DOI:** 10.1107/S1600536813005692

**Published:** 2013-03-06

**Authors:** Muhammad Taha, Nor Hadiani Ismail, Faridahanim Mohd Jaafar, Ahmad Nazif Aziz, Sammer Yousuf

**Affiliations:** a Atta-ur-Rahman Institute for Natural Product Discovery, Universiti Teknologi MARA (UiTM), Puncak Alam Campus, 42300 Bandar Puncak Alam, Selangor D. E., Malaysia; b Faculty of Applied Science, Universiti Teknologi MARA (UiTM), 40450 Shah Alam, Selangor D. E., Malaysia; c Department of Chemical Sciences, Faculty of Science and Technology, University Malaysia Terengganu, 21030, Kuala Terengganu, Malaysia; d H.E.J. Research Institute of Chemistry, International Center for Chemical and Biological Sciences, University of Karachi, Karachi 75270, Pakistan

## Abstract

In the title compound, C_16_H_16_N_2_O_3_·H_2_O, the dihedral angle between the benzene rings is 30.27 (7)°. In the crystal, the components are linked by N—H⋯O, O—H⋯O and C—H⋯O inter­actions into a three-dimensional network.

## Related literature

For the applications and biological activity of Schiff bases, see: Musharraf *et al.* (2012 ▶); Khan *et al.* (2012 ▶). For the crystal structures of related compounds, see: Taha *et al.* (2012 ▶); Baharudin *et al.* (2012 ▶).
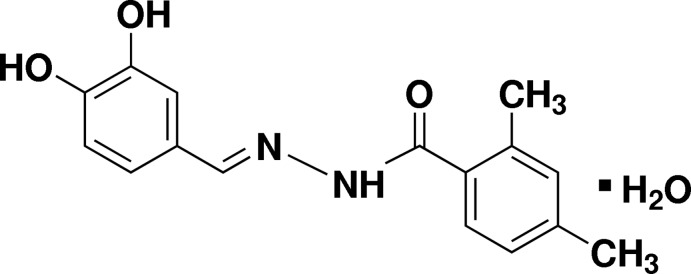


## Experimental

### 

#### Crystal data

C_16_H_16_N_2_O_3_·H_2_O
*M* *_r_* = 302.32
Monoclinic, 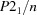

*a* = 8.1373 (3) Å
*b* = 13.9025 (5) Å
*c* = 13.7886 (5) Å
β = 92.913 (1)°
*V* = 1557.87 (10) Å^3^
*Z* = 4
Mo *K*α radiation
μ = 0.09 mm^−1^
*T* = 298 K
0.30 × 0.10 × 0.10 mm

#### Data collection

Bruker SMART APEX CCD area-detector diffractometer
Absorption correction: multi-scan (*SADABS*; Bruker, 2000 ▶) *T*_min_ = 0.973, *T*_max_ = 0.991
9012 measured reflections
2897 independent reflections
2535 reflections with *I* > 2σ(*I*)
*R*_int_ = 0.016

#### Refinement

*R*[*F* ^2^ > 2σ(*F* ^2^)] = 0.040
*wR*(*F* ^2^) = 0.111
*S* = 1.05
2897 reflections
221 parameters
H atoms treated by a mixture of independent and constrained refinement
Δρ_max_ = 0.19 e Å^−3^
Δρ_min_ = −0.28 e Å^−3^

### 

Data collection: *SMART* (Bruker, 2000 ▶); cell refinement: *SAINT* (Bruker, 2000 ▶); data reduction: *SAINT*; program(s) used to solve structure: *SHELXS97* (Sheldrick, 2008 ▶); program(s) used to refine structure: *SHELXL97* (Sheldrick, 2008 ▶); molecular graphics: *SHELXTL* (Sheldrick, 2008 ▶); software used to prepare material for publication: *SHELXTL*, *PARST* (Nardelli, 1995 ▶) and *PLATON* (Spek, 2009 ▶).

## Supplementary Material

Crystal structure: contains datablock(s) global, I. DOI: 10.1107/S1600536813005692/pv2622sup1.cif

Structure factors: contains datablock(s) I. DOI: 10.1107/S1600536813005692/pv2622Isup2.hkl

Supplementary material file. DOI: 10.1107/S1600536813005692/pv2622Isup3.cml

Additional supplementary materials:  crystallographic information; 3D view; checkCIF report

## Figures and Tables

**Table 1:** Hydrogen-bond geometry (Å, °)

*D*—H⋯*A*	*D*—H	H⋯*A*	*D*⋯*A*	*D*—H⋯*A*
O1*W*—H1*W*1⋯N2^i^	0.87 (2)	2.20 (2)	3.059 (2)	169 (2)
O1*W*—H2*W*1⋯O1	0.94 (2)	2.01 (2)	2.935 (2)	173 (2)
N1—H1*A*⋯O3^ii^	0.91 (2)	2.08 (2)	2.962 (2)	163 (2)
O2—H2*A*⋯O1^i^	0.88 (2)	1.94 (2)	2.791 (2)	162 (2)
O3—H3*A*⋯O1*W* ^iii^	0.85 (2)	1.79 (2)	2.629 (2)	172 (2)
C8—H8*A*⋯O3^ii^	0.93	2.58	3.382 (2)	145
C15—H15*B*⋯O2^i^	0.96	2.52	3.351 (2)	144

Symmetry codes: (i) 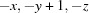; (ii) 
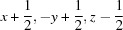
; (iii) 
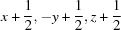
.

**Fig. 1.**
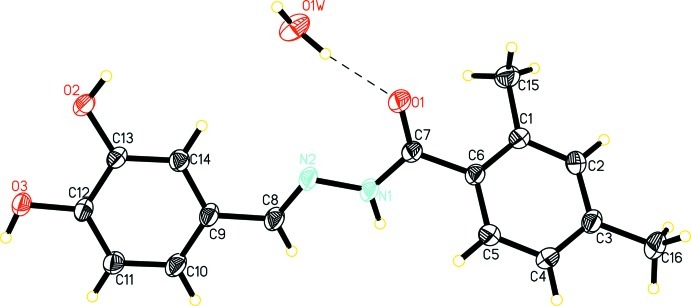
The molecular structure of the title compound with displacement ellipsoids drawn at 30% probability level.

**Fig. 2.**
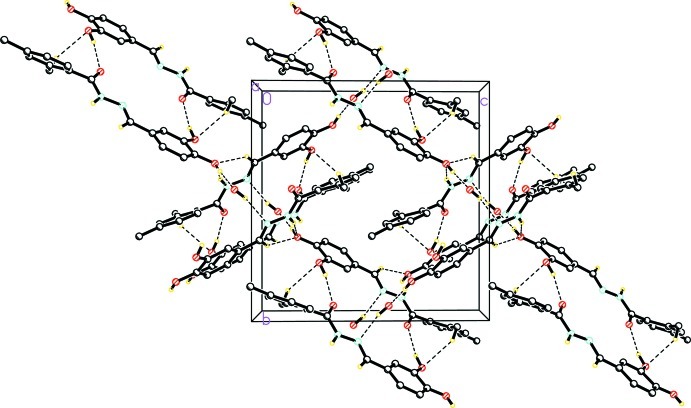
The crystal packing of the title compound. Only hydrogen atoms involved in hydrogen bonding are shown.

## supplementary crystallographic information

### Comment

Structurally diverse range of benzohydrazides have been extensively studied in order to explore the structural features that may be responsible for different biological activities (Musharraf *et al.*, 2012; Khan *et al.*, 2012). The title compound is yet another benzohydrazide monohydrate, obtained as a part of our ongoing research that has been studied by X-ray crystallographic method and reported in this article.

In the title compound (Fig. 1) dimethyl and dihydroxy substituted benzene rings (C1–C6 and C9–C14, respectively) are each planner with a dihedral angle 30.27 (7)° between their mean-planes. The azomethine double bond, N2═C8 (1.2729 (19) Å) adopts an *E* configuration. The bond lengths and angle are similar to the corresponding bond lengths and angles reported in structurally related benzohydrazide derivatives (Taha *et al.*, 2012; Baharudin *et al.*, 2012). The crystal structure is stabilized by N1—H1A···N2, O2—H2A···O1, C8—H8A···O3 and C15—H15B···O2 intermolecular interactions. The ineteractions further extend the structure to a three dimentional network *via* O1W—H2W1···O1, O1W—H1A···O3 and O3—H3A···O1W interactions involving the water of hydration (Table 2 and Fig. 2).

### Experimental

The title compound was synthesized by reacting (0.328 g, 2 mmol) 2,4-dimethylbenzohydrazide and (0.276 g, 2 mmol) 3,4-dihydroxybenzaldehyde as starting meterial under the same conditions and solvents as described previously for the synthesis of benzohydrazides (Taha *et al.*, 2012). The title compound was recrystalized by dissolving in methanol to obtain colorless needles (0.499 g, 88% yield). All chemicals were purchased by Sigma Aldrich Germany.

### Refinement

H atoms on methyl and benzene ring were positioned geometrically with C—H = 0.96 and 0.93 Å, respectively and constrained to ride on their parent atoms with *U*_iso_(H) = 1.2*U*_eq_(benzene) or 1.5*U*_eq_(methyl). The H atoms on oxygen and nitrogen were located in difference Fourier map and refined isotropically. A rotating group model was applied to the methyl groups.

### Crystal data

**Table:**

C_16_H_16_N_2_O_3_·H_2_O	*F*(000) = 640
*M**_r_* = 302.32	*D*_x_ = 1.289 Mg m^−^^3^
Monoclinic, *P*2_1_/*n*	Mo *K*α radiation, λ = 0.71073 Å
Hall symbol: -P 2yn	Cell parameters from 4856 reflections
*a* = 8.1373 (3) Å	θ = 2.8–28.2°
*b* = 13.9025 (5) Å	µ = 0.09 mm^−^^1^
*c* = 13.7886 (5) Å	*T* = 298 K
β = 92.913 (1)°	Block, brown
*V* = 1557.87 (10) Å^3^	0.30 × 0.10 × 0.10 mm
*Z* = 4	

### Data collection

**Table:**

Bruker SMART APEX CCD area-detector diffractometer	2897 independent reflections
Radiation source: fine-focus sealed tube	2535 reflections with *I* > 2σ(*I*)
Graphite monochromator	*R*_int_ = 0.016
ω scan	θ_max_ = 25.5°, θ_min_ = 2.1°
Absorption correction: multi-scan (*SADABS*; Bruker, 2000)	*h* = −9→9
*T*_min_ = 0.973, *T*_max_ = 0.991	*k* = −16→16
9012 measured reflections	*l* = −16→13

### Refinement

**Table:**

Refinement on *F*^2^	Primary atom site location: structure-invariant direct methods
Least-squares matrix: full	Secondary atom site location: difference Fourier map
*R*[*F*^2^ > 2σ(*F*^2^)] = 0.040	Hydrogen site location: inferred from neighbouring sites
*wR*(*F*^2^) = 0.111	H atoms treated by a mixture of independent and constrained refinement
*S* = 1.05	*w* = 1/[σ^2^(*F*_o_^2^) + (0.0596*P*)^2^ + 0.3352*P*] where *P* = (*F*_o_^2^ + 2*F*_c_^2^)/3
2897 reflections	(Δ/σ)_max_ < 0.001
221 parameters	Δρ_max_ = 0.19 e Å^−^^3^
0 restraints	Δρ_min_ = −0.28 e Å^−^^3^

### Special details

**Table:**

Geometry. All e.s.d.'s (except the e.s.d. in the dihedral angle between two l.s. planes) are estimated using the full covariance matrix. The cell e.s.d.'s are taken into account individually in the estimation of e.s.d.'s in distances, angles and torsion angles; correlations between e.s.d.'s in cell parameters are only used when they are defined by crystal symmetry. An approximate (isotropic) treatment of cell e.s.d.'s is used for estimating e.s.d.'s involving l.s. planes.
Refinement. Refinement of *F*^2^ against ALL reflections. The weighted *R*-factor *wR* and goodness of fit *S* are based on *F*^2^, conventional *R*-factors *R* are based on *F*, with *F* set to zero for negative *F*^2^. The threshold expression of *F*^2^ > σ(*F*^2^) is used only for calculating *R*-factors(gt) *etc*. and is not relevant to the choice of reflections for refinement. *R*-factors based on *F*^2^ are statistically about twice as large as those based on *F*, and *R*- factors based on ALL data will be even larger.

### Fractional atomic coordinates and isotropic or equivalent isotropic displacement parameters (Å^2^)

**Table:**

	*x*	*y*	*z*	*U*_iso_*/*U*_eq_	
O1	0.15363 (13)	0.55077 (8)	−0.16328 (8)	0.0569 (3)	
O1W	−0.12355 (15)	0.45259 (10)	−0.07769 (9)	0.0612 (3)	
O2	−0.04800 (12)	0.26761 (9)	0.22605 (8)	0.0505 (3)	
O3	0.16469 (13)	0.15980 (8)	0.32684 (7)	0.0460 (3)	
N1	0.34634 (15)	0.43523 (9)	−0.14057 (9)	0.0454 (3)	
N2	0.28104 (14)	0.40196 (9)	−0.05580 (8)	0.0435 (3)	
C1	0.30375 (16)	0.56844 (10)	−0.36100 (10)	0.0395 (3)	
C2	0.40413 (18)	0.60048 (11)	−0.43273 (10)	0.0446 (3)	
H2C	0.3561	0.6153	−0.4935	0.054*	
C3	0.57324 (17)	0.61142 (11)	−0.41767 (10)	0.0435 (3)	
C4	0.64410 (17)	0.58608 (11)	−0.32796 (11)	0.0455 (4)	
H4A	0.7574	0.5911	−0.3166	0.055*	
C5	0.54846 (16)	0.55358 (10)	−0.25556 (10)	0.0423 (3)	
H5A	0.5981	0.5371	−0.1956	0.051*	
C6	0.37828 (16)	0.54484 (9)	−0.27016 (10)	0.0366 (3)	
C7	0.28131 (16)	0.51150 (10)	−0.18789 (10)	0.0397 (3)	
C8	0.35766 (18)	0.33077 (11)	−0.01702 (11)	0.0464 (4)	
H8A	0.4495	0.3069	−0.0465	0.056*	
C9	0.30656 (17)	0.28538 (10)	0.07190 (10)	0.0421 (3)	
C10	0.41516 (19)	0.22570 (11)	0.12370 (12)	0.0514 (4)	
H10A	0.5192	0.2145	0.1011	0.062*	
C11	0.37047 (19)	0.18256 (11)	0.20869 (12)	0.0499 (4)	
H11A	0.4448	0.1431	0.2432	0.060*	
C12	0.21552 (16)	0.19792 (9)	0.24262 (10)	0.0390 (3)	
C13	0.10379 (16)	0.25680 (10)	0.18973 (10)	0.0376 (3)	
C14	0.14912 (16)	0.29972 (10)	0.10553 (10)	0.0392 (3)	
H14A	0.0745	0.3387	0.0705	0.047*	
C15	0.12199 (18)	0.55713 (14)	−0.38483 (12)	0.0571 (4)	
H15A	0.1029	0.5506	−0.4538	0.086*	
H15B	0.0648	0.6128	−0.3628	0.086*	
H15C	0.0825	0.5008	−0.3531	0.086*	
C16	0.6765 (2)	0.65068 (14)	−0.49624 (13)	0.0615 (5)	
H16A	0.6324	0.6293	−0.5584	0.092*	
H16B	0.7875	0.6280	−0.4863	0.092*	
H16C	0.6755	0.7197	−0.4941	0.092*	
H2A	−0.099 (3)	0.3180 (16)	0.1996 (15)	0.077 (6)*	
H1A	0.436 (2)	0.4053 (13)	−0.1634 (13)	0.061 (5)*	
H1W1	−0.165 (3)	0.4883 (17)	−0.0335 (16)	0.080 (7)*	
H3A	0.239 (2)	0.1248 (15)	0.3533 (15)	0.071 (6)*	
H2W1	−0.029 (3)	0.4798 (17)	−0.1022 (16)	0.091 (7)*	

### Atomic displacement parameters (Å^2^)

**Table:**

	*U*^11^	*U*^22^	*U*^33^	*U*^12^	*U*^13^	*U*^23^
O1	0.0495 (6)	0.0633 (7)	0.0601 (7)	0.0209 (5)	0.0261 (5)	0.0182 (5)
O1W	0.0486 (6)	0.0766 (9)	0.0595 (7)	−0.0124 (6)	0.0122 (5)	−0.0274 (6)
O2	0.0399 (5)	0.0578 (7)	0.0557 (7)	0.0085 (5)	0.0200 (5)	0.0140 (5)
O3	0.0453 (6)	0.0499 (6)	0.0442 (6)	0.0056 (5)	0.0153 (5)	0.0141 (5)
N1	0.0462 (6)	0.0506 (7)	0.0414 (7)	0.0134 (6)	0.0227 (5)	0.0112 (5)
N2	0.0453 (6)	0.0477 (7)	0.0393 (6)	0.0066 (5)	0.0194 (5)	0.0077 (5)
C1	0.0360 (7)	0.0429 (7)	0.0400 (7)	0.0004 (5)	0.0051 (6)	0.0011 (6)
C2	0.0478 (8)	0.0505 (8)	0.0358 (7)	0.0008 (6)	0.0041 (6)	0.0062 (6)
C3	0.0437 (7)	0.0430 (8)	0.0448 (8)	−0.0010 (6)	0.0136 (6)	0.0037 (6)
C4	0.0335 (7)	0.0506 (8)	0.0530 (9)	−0.0033 (6)	0.0068 (6)	0.0038 (7)
C5	0.0383 (7)	0.0488 (8)	0.0396 (7)	0.0023 (6)	0.0014 (6)	0.0048 (6)
C6	0.0360 (7)	0.0379 (7)	0.0367 (7)	0.0023 (5)	0.0085 (5)	0.0023 (5)
C7	0.0378 (7)	0.0434 (7)	0.0387 (7)	0.0050 (6)	0.0105 (6)	0.0030 (6)
C8	0.0484 (8)	0.0466 (8)	0.0463 (8)	0.0108 (7)	0.0218 (6)	0.0064 (7)
C9	0.0468 (8)	0.0393 (7)	0.0418 (7)	0.0055 (6)	0.0176 (6)	0.0046 (6)
C10	0.0468 (8)	0.0526 (9)	0.0572 (9)	0.0149 (7)	0.0263 (7)	0.0122 (7)
C11	0.0469 (8)	0.0496 (9)	0.0547 (9)	0.0156 (7)	0.0171 (7)	0.0154 (7)
C12	0.0441 (7)	0.0352 (7)	0.0388 (7)	0.0008 (6)	0.0145 (6)	0.0039 (5)
C13	0.0370 (7)	0.0365 (7)	0.0405 (7)	0.0007 (5)	0.0128 (5)	−0.0002 (5)
C14	0.0420 (7)	0.0361 (7)	0.0401 (7)	0.0036 (6)	0.0082 (6)	0.0034 (6)
C15	0.0401 (8)	0.0788 (12)	0.0521 (9)	−0.0036 (7)	−0.0010 (7)	0.0037 (8)
C16	0.0567 (9)	0.0699 (11)	0.0598 (10)	−0.0030 (8)	0.0225 (8)	0.0146 (9)

### Geometric parameters (Å, º)

**Table:**

O1—C7	1.2364 (16)	C5—C6	1.3945 (19)
O1W—H1W1	0.87 (2)	C5—H5A	0.9300
O1W—H2W1	0.94 (2)	C6—C7	1.4884 (18)
O2—C13	1.3645 (15)	C8—C9	1.4581 (19)
O2—H2A	0.88 (2)	C8—H8A	0.9300
O3—C12	1.3599 (16)	C9—C10	1.384 (2)
O3—H3A	0.85 (2)	C9—C14	1.3988 (19)
N1—C7	1.3398 (18)	C10—C11	1.382 (2)
N1—N2	1.3877 (15)	C10—H10A	0.9300
N1—H1A	0.913 (19)	C11—C12	1.3835 (19)
N2—C8	1.2729 (19)	C11—H11A	0.9300
C1—C2	1.3878 (19)	C12—C13	1.4004 (19)
C1—C6	1.4026 (19)	C13—C14	1.3725 (19)
C1—C15	1.5069 (19)	C14—H14A	0.9300
C2—C3	1.390 (2)	C15—H15A	0.9600
C2—H2C	0.9300	C15—H15B	0.9600
C3—C4	1.384 (2)	C15—H15C	0.9600
C3—C16	1.507 (2)	C16—H16A	0.9600
C4—C5	1.373 (2)	C16—H16B	0.9600
C4—H4A	0.9300	C16—H16C	0.9600
			
H1W1—O1W—H2W1	112 (2)	C10—C9—C14	119.07 (13)
C13—O2—H2A	110.5 (13)	C10—C9—C8	119.44 (12)
C12—O3—H3A	110.4 (13)	C14—C9—C8	121.48 (13)
C7—N1—N2	121.02 (11)	C11—C10—C9	120.62 (13)
C7—N1—H1A	119.8 (11)	C11—C10—H10A	119.7
N2—N1—H1A	119.2 (11)	C9—C10—H10A	119.7
C8—N2—N1	114.37 (11)	C10—C11—C12	120.24 (14)
C2—C1—C6	117.91 (12)	C10—C11—H11A	119.9
C2—C1—C15	119.00 (13)	C12—C11—H11A	119.9
C6—C1—C15	123.05 (12)	O3—C12—C11	123.41 (13)
C1—C2—C3	122.88 (13)	O3—C12—C13	117.06 (11)
C1—C2—H2C	118.6	C11—C12—C13	119.52 (12)
C3—C2—H2C	118.6	O2—C13—C14	123.35 (12)
C4—C3—C2	118.05 (13)	O2—C13—C12	116.69 (12)
C4—C3—C16	120.85 (13)	C14—C13—C12	119.96 (12)
C2—C3—C16	121.10 (13)	C13—C14—C9	120.57 (13)
C5—C4—C3	120.53 (13)	C13—C14—H14A	119.7
C5—C4—H4A	119.7	C9—C14—H14A	119.7
C3—C4—H4A	119.7	C1—C15—H15A	109.5
C4—C5—C6	121.27 (13)	C1—C15—H15B	109.5
C4—C5—H5A	119.4	H15A—C15—H15B	109.5
C6—C5—H5A	119.4	C1—C15—H15C	109.5
C5—C6—C1	119.32 (12)	H15A—C15—H15C	109.5
C5—C6—C7	118.57 (12)	H15B—C15—H15C	109.5
C1—C6—C7	122.11 (12)	C3—C16—H16A	109.5
O1—C7—N1	122.17 (12)	C3—C16—H16B	109.5
O1—C7—C6	123.86 (12)	H16A—C16—H16B	109.5
N1—C7—C6	113.96 (11)	C3—C16—H16C	109.5
N2—C8—C9	122.36 (12)	H16A—C16—H16C	109.5
N2—C8—H8A	118.8	H16B—C16—H16C	109.5
C9—C8—H8A	118.8		
			
C7—N1—N2—C8	−178.10 (14)	C5—C6—C7—N1	−45.73 (18)
C6—C1—C2—C3	−1.1 (2)	C1—C6—C7—N1	134.90 (14)
C15—C1—C2—C3	−178.87 (15)	N1—N2—C8—C9	−179.45 (13)
C1—C2—C3—C4	2.2 (2)	N2—C8—C9—C10	−163.72 (16)
C1—C2—C3—C16	−177.21 (15)	N2—C8—C9—C14	17.1 (2)
C2—C3—C4—C5	−1.7 (2)	C14—C9—C10—C11	−1.5 (2)
C16—C3—C4—C5	177.66 (15)	C8—C9—C10—C11	179.34 (15)
C3—C4—C5—C6	0.2 (2)	C9—C10—C11—C12	0.6 (3)
C4—C5—C6—C1	0.9 (2)	C10—C11—C12—O3	−178.29 (14)
C4—C5—C6—C7	−178.49 (13)	C10—C11—C12—C13	0.6 (2)
C2—C1—C6—C5	−0.5 (2)	O3—C12—C13—O2	−2.27 (19)
C15—C1—C6—C5	177.23 (14)	C11—C12—C13—O2	178.79 (14)
C2—C1—C6—C7	178.88 (13)	O3—C12—C13—C14	178.14 (12)
C15—C1—C6—C7	−3.4 (2)	C11—C12—C13—C14	−0.8 (2)
N2—N1—C7—O1	−5.9 (2)	O2—C13—C14—C9	−179.69 (13)
N2—N1—C7—C6	172.84 (12)	C12—C13—C14—C9	−0.1 (2)
C5—C6—C7—O1	133.03 (16)	C10—C9—C14—C13	1.3 (2)
C1—C6—C7—O1	−46.3 (2)	C8—C9—C14—C13	−179.59 (14)

### Hydrogen-bond geometry (Å, º)

**Table:**

*D*—H···*A*	*D*—H	H···*A*	*D*···*A*	*D*—H···*A*
O1*W*—H1*W*1···N2^i^	0.87 (2)	2.20 (2)	3.059 (2)	169 (2)
N1—H1*A*···O3^ii^	0.91 (2)	2.08 (2)	2.962 (2)	163 (2)
O1*W*—H2*W*1···O1	0.94 (2)	2.01 (2)	2.935 (2)	173 (2)
O2—H2*A*···O1^i^	0.88 (2)	1.94 (2)	2.791 (2)	162 (2)
O3—H3*A*···O1*W*^iii^	0.85 (2)	1.79 (2)	2.629 (2)	172 (2)
C8—H8*A*···O3^ii^	0.93	2.58	3.382 (2)	145
C15—H15*B*···O2^i^	0.96	2.52	3.351 (2)	144

Symmetry codes: (i) −*x*, −*y*+1, −*z*; (ii) *x*+1/2, −*y*+1/2, *z*−1/2; (iii) *x*+1/2, −*y*+1/2, *z*+1/2.

## References

[bb1] Baharudin, M. S., Taha, M., Ismail, N. H., Shah, S. A. A. & Yousuf, S. (2012). *Acta Cryst.* E**68**, o3255. 10.1107/S1600536812042389 PMC3588809 23468774

[bb2] Bruker (2000). *SADABS*, *SMART* and *SAINT* Bruker AXS Inc., Madison, Wisconsin, USA.

[bb3] Khan, K. M., Taha, M., Naz, F., Siddiqui, S., Rahim, F., Perveen, S. & Choudhary, M. I. (2012). *Med. Chem.* **8**, 705–710. 10.2174/157340612801216111 22571188

[bb4] Musharraf, S. G., Bibi, A., Shahid, N., Najam-ul-Haq, M., Khan, M., Taha, M., Mughal, U. R. & Khan, K. M. (2012). *Am. J. Anal. Chem.* **3**, 779-789.

[bb5] Nardelli, M. (1995). *J. Appl. Cryst.* **28**, 659.

[bb6] Sheldrick, G. M. (2008). *Acta Cryst.* A**64**, 112–122. 10.1107/S0108767307043930 18156677

[bb7] Spek, A. L. (2009). *Acta Cryst.* D**65**, 148–155. 10.1107/S090744490804362X PMC2631630 19171970

[bb8] Taha, M., Naz, H., Rahman, A. A., Ismail, N. H. & Sammer, Y. (2012). *Acta Cryst.* E**68**, o2778. 10.1107/S1600536812034988 PMC3435810 22969656

